# Cytomegalovirus Corneal Endotheliitis Presenting As Bullous Keratopathy: Resolution With Antiviral Therapy Alone

**DOI:** 10.7759/cureus.84175

**Published:** 2025-05-15

**Authors:** Koichi Sakamoto, Hideki Fukuoka, Yohei Otsuki, Chie Sotozono

**Affiliations:** 1 Ophthalmology, Kyoto Prefectural University of Medicine, Kyoto, JPN; 2 Ophthalmology, Matsushita Memorial Hospital, Osaka, JPN

**Keywords:** aqueous humor pcr, bullous keratopathy, corneal endotheliitis, cytomegalovirus (cmv), ganciclovir

## Abstract

Bullous keratopathy is a condition that arises from corneal endothelial dysfunction, leading to corneal edema. Corneal transplantation has historically served as the standard treatment for cases with severe visual impairment due to edema, given the generally accepted irreversible nature of the condition. The present case report documents the successful management of a patient with steroid-resistant corneal edema through administering anti-cytomegalovirus (CMV) therapy, thereby underscoring the potential for alternative treatment modalities in cases of visual impairment due to corneal edema. A 73-year-old male patient presented at our clinic with steroid-resistant corneal edema. He had a 10-year history of recurrent right corneal endotheliitis. A thorough clinical evaluation revealed substantial corneal edema in the right eye, accompanied by an initial best-corrected visual acuity of 0.06 (20/333), which remained uncorrected by conventional optical aids. We proceeded with aqueous humor analysis via viral polymerase chain reaction testing, which revealed CMV positivity (while herpes simplex virus and varicella-zoster virus testing were negative). Consequently, the patient was diagnosed with CMV-related corneal endotheliitis, and treatment with 0.5% ganciclovir eye drops was initiated. Following treatment, a significant improvement in the corneal edema was observed, and the patient's best-corrected visual acuity recovered to 0.6-1.0 (20/33-20/20). This clinical experience underscores the significance of molecular diagnostic approaches in ophthalmic practice, illustrating how targeted antiviral therapy can effectively restore corneal function in cases previously regarded as suitable only for surgical intervention. Sustained visual improvement was documented throughout the one-year follow-up period, suggesting a paradigm shift in managing select cases of bullous keratopathy.

## Introduction

Bullous keratopathy, a condition characterized by fluid-filled blisters and swelling in the cornea due to failure of the endothelial cell pump function, is caused by irreversible and permanent damage to the corneal endothelium, leading to edema of the corneal stroma and epithelium. The function of the endothelial cells' pump is critical for maintaining a constant water content in the corneal stroma, thereby ensuring the regular arrangement of collagen fibers in the stroma and preserving corneal transparency [1]. However, due to the absence of regenerative capacity in vivo, the functional decline resulting from bullous keratopathy is irreversible, leading to corneal opacity due to epithelial and stromal edema and subsequent vision impairment. The therapeutic interventions for this condition include pharmacological therapy, such as activating endothelial cells with adrenocortical steroids, using hypertonic saline to induce dehydration, and corneal endothelial transplantation to replenish endothelial cells. Other treatments to alleviate pain include therapeutic contact lenses, phototherapeutic keratectomy, and amniotic membrane transplantation.

Cytomegalovirus (CMV) corneal endotheliitis, a form of inflammation affecting the innermost layer of the cornea (endothelium), typically presents unilaterally in middle-aged to elderly immunocompetent men without underlying systemic conditions [2]. It has recently garnered attention as an underrecognized and underestimated cause of bullous keratopathy. Additionally, polymerase chain reaction (PCR) testing of aqueous humor is a useful diagnostic tool for identifying the infectious etiology of this condition.

For CMV corneal endotheliitis, local treatment with ganciclovir eye drops, steroid eye drops, and systemic treatment with ganciclovir infusion or its prodrug valganciclovir orally are considered effective. Initial treatment regimens may include systemic therapy with ganciclovir 5 mg/kg twice daily for two weeks by infusion, or valganciclovir 900 mg twice daily for four to 12 weeks orally, or local treatment with 0.5% ganciclovir eye drops four to eight times daily and 0.1% fluorometholone four times daily [2]. While there are some reports of improvement in mild visual impairment and corneal edema with anti-CMV treatment [3], traditionally, severe cases with extensive edema and significant visual impairment have generally required surgical intervention such as corneal endothelial cell transplantation.

This report presents a remarkable case that challenges conventional treatment paradigms. A patient with bullous keratopathy exhibiting extensive stromal edema and pronounced vision loss was successfully treated with pharmacological therapy alone. The patient's diagnosis was confirmed through the analysis of aqueous humor using a PCR technique to detect CMV. The patient exhibited a substantial improvement in both corneal edema and visual acuity following treatment with 0.5% ganciclovir eye drops, thereby obviating the necessity for surgical intervention.

## Case presentation

A 73-year-old male patient was referred to the Department of Ophthalmology at Kyoto Prefectural University of Medicine (referral day). The patient had been under the care of the ophthalmology department at Hospital A for recurrent right keratitis for a decade. The inflammation had typically been managed with steroid eye drops during these recurrences. However, the patient developed steroid-resistant corneal edema and elevated intraocular pressure (21-22 mmHg), which was being managed with multiple glaucoma drops (as detailed below). These findings led to the patient's referral to the ophthalmology department at Kyoto Prefectural University of Medicine for surgical management of bullous keratopathy.

The patient's medical history was notable for ongoing oral steroid treatment for interstitial pneumonia, prior treatment for bilateral idiopathic femoral head necrosis, and the presence of colon polyps. His ocular history included right endotheliitis with documented flare-ups 10 years before referral, two years before referral, and one year before referral, bilateral glaucoma diagnosed six years before referral, and bilateral cataract surgery performed three years before referral.

**Figure 1:**
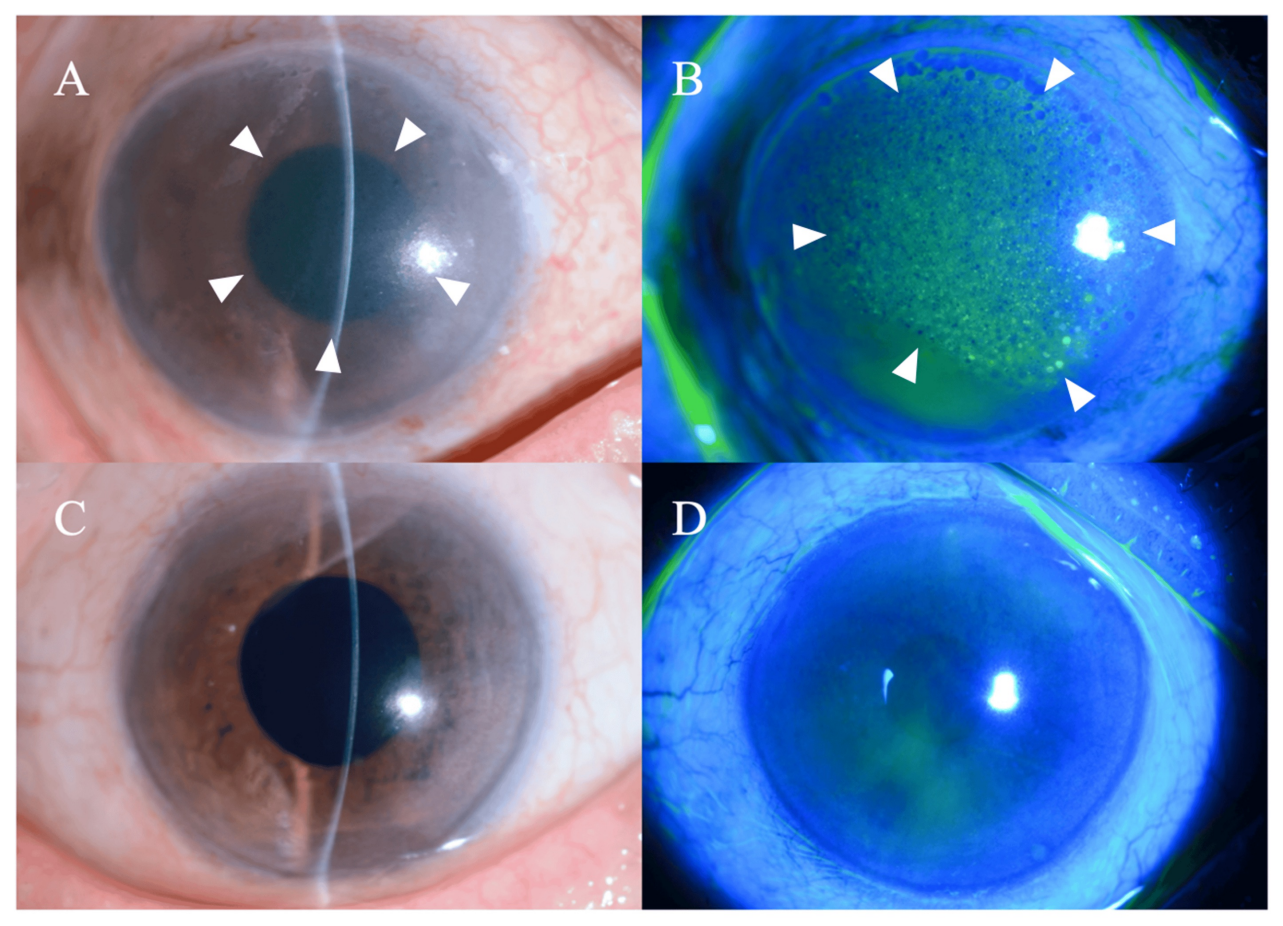
Slit-lamp findings of the anterior segment at the initial visit (A) Right eye showing significant corneal edema with internal structures not visible (white arrowheads). (B) Right eye with fluorescein staining showing diffuse punctate epithelial keratopathy (white arrowheads). (C) Left eye showing no abnormal findings with a clear cornea. (D) Left eye with fluorescein staining showing no abnormal staining pattern.

A thorough examination revealed best-corrected visual acuity of 0.06 (20/333) in the right eye and 0.8 (20/25) in the left eye. Intraocular pressure was not measured in the right eye, and it was 19.7 mmHg in the left eye. Anterior segment examination revealed significant corneal edema in the right eye (Figure 1A-1B), with internal structures not visible, while the left eye showed no abnormal findings (Figure 1C-1D).

**Figure 2:**
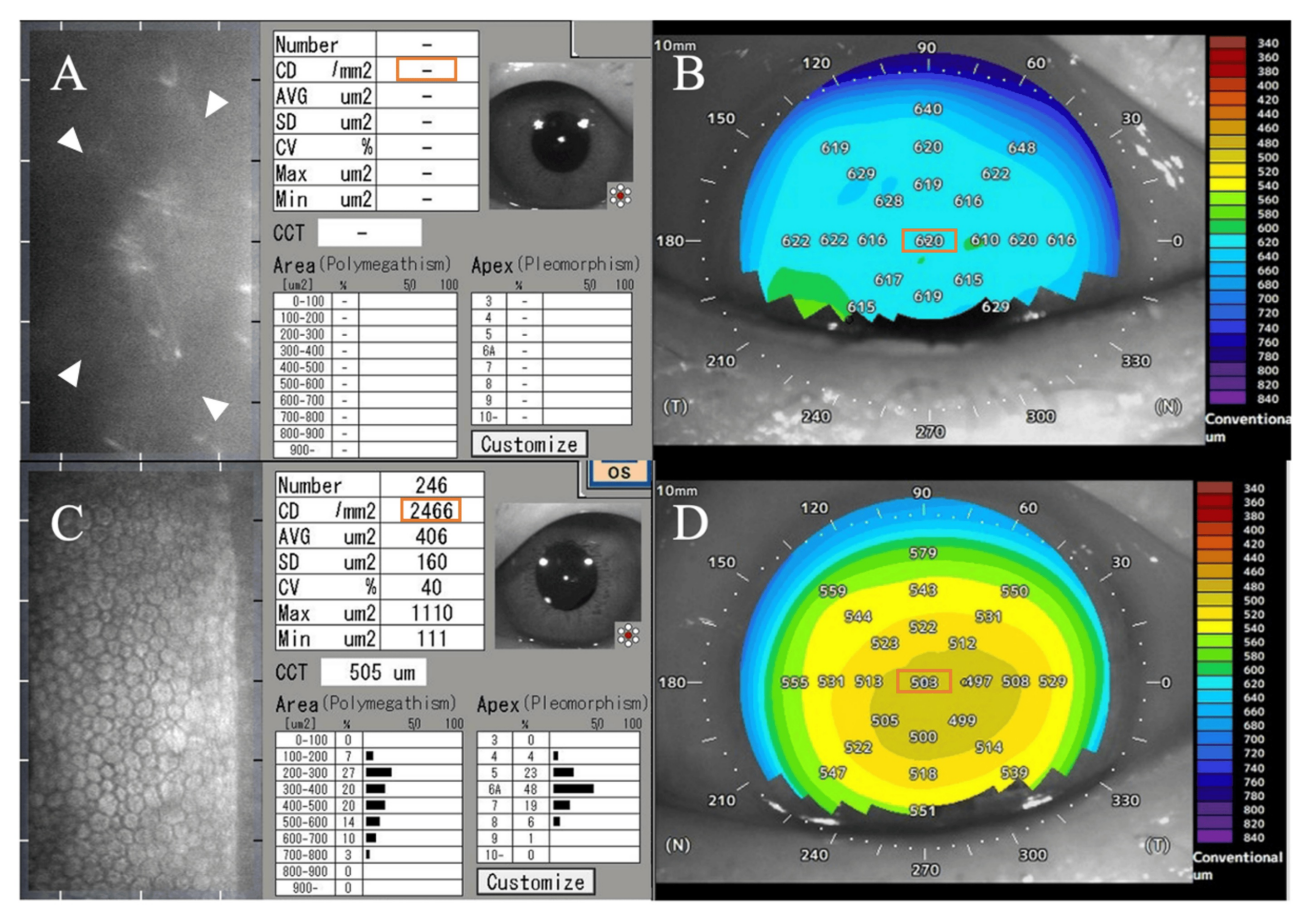
Endothelial specular microscopy and anterior segment OCT findings at initial visit (A) Specular microscopy of the right eye was not possible due to severe corneal edema (white arrowheads), and cell density could not be measured (orange rectangle). (B) Anterior segment OCT showed diffuse thickening of the right cornea with a central thickness of 620 μm (orange rectangle), as seen in the pachymetry map. (C) The left eye showed a normal endothelial mosaic with a cell density of 2466 cells/mm² (orange rectangle) on specular microscopy. (D) Anterior segment OCT of the left eye revealed a normal central corneal thickness of 503 μm (orange rectangle), within the normal range on the pachymetry map. OCT: optical coherence tomography

Endothelial specular microscopy could not be performed on the right eye due to corneal edema (Figure 2A). Anterior segment optical coherence tomography (OCT) (CASIA2, Tomey Corporation, Nagoya, Japan) revealed diffuse thickening of the right cornea with a central corneal thickness of 620 μm (Figure 2B). In contrast, the left eye exhibited an endothelial cell density of 2466 cells/mm² (Figure 2C). The left cornea showed normal findings on anterior segment OCT with a central corneal thickness of 503 μm (Figure 2D).

During the preliminary visit, the patient was found to be using the following medications: betamethasone sodium phosphate (0.1% Rinderon® eye drops, right eye four times per day), latanoprost (0.05% Xalatan® eye drops, right eye once per day), dorzolamide hydrochloride/timolol maleate (Cosopt® combination eye drops, both eyes twice per day), and ripasudil hydrochloride hydrate/brimonidine tartrate (Glaalfa® combination eye drops, right eye twice per day).

**Figure 3:**
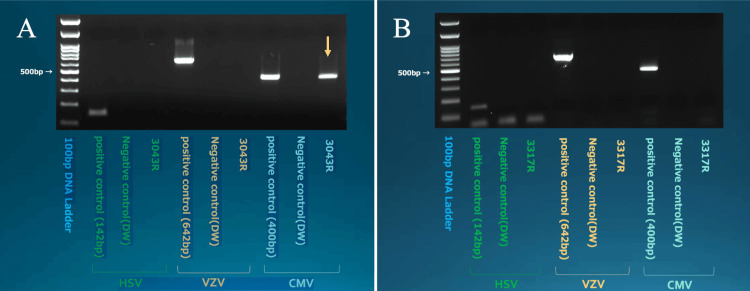
Qualitative PCR results of aqueous humor for herpesvirus detection at referral and 13-month follow-up (A) Aqueous humor PCR testing was negative for HSV and VZV but positive for CMV (yellow arrow) at referral. Sample 3043R represents the aqueous humor from the right eye at the time of referral. (B) Aqueous humor testing was performed again 13 months after referral, confirming the negative for CMV. Sample 3317R represents the aqueous humor from the right eye at 18 months follow-up. PCR: polymerase chain reaction, HSV: herpes simplex virus, VZV: varicella-zoster virus, CMV: cytomegalovirus

Aqueous humor PCR testing was negative for herpes simplex virus (HSV) and varicella-zoster virus (VZV) but positive for CMV (Figure 3A). The PCR method used was conventional gel-based PCR, and viral detection was qualitative, not quantitative. In light of these clinical findings and laboratory results, the patient was diagnosed with bullous keratopathy due to CMV corneal endotheliitis.

Seven days after referral, the treatment regimen was modified. The patient was administered 0.5% ganciclovir eye drops (hospital-compounded, right eye, eight times per day), while betamethasone sodium phosphate eye drops were reduced to once daily in the right eye. The patient was instructed to continue using dorzolamide hydrochloride/timolol maleate eye drops (both eyes twice per day), and latanoprost eye drops were discontinued. Additionally, ripasudil hydrochloride hydrate/brimonidine tartrate eye drops were discontinued.

**Figure 4:**
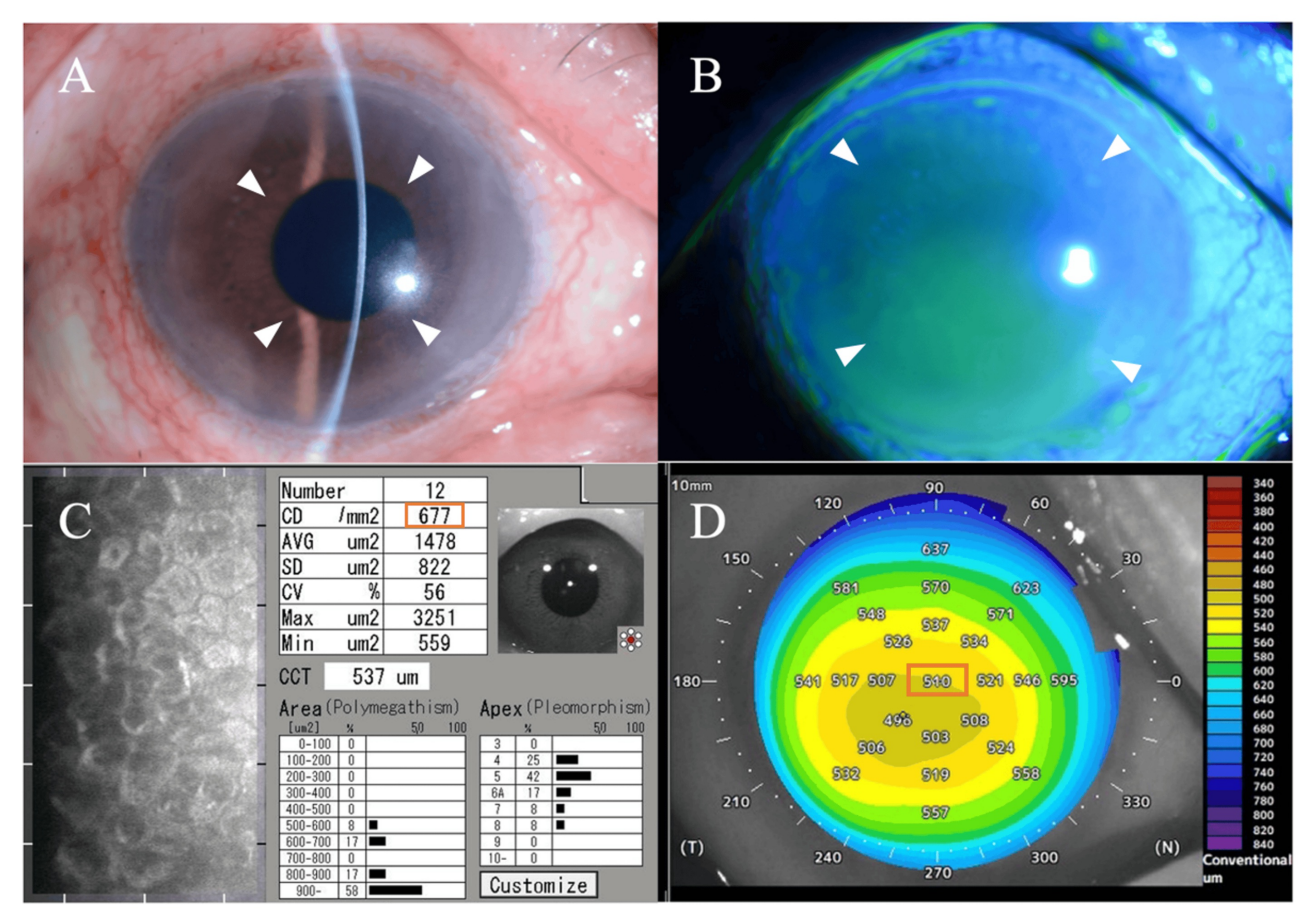
Anterior segment findings 13 months after pharmacological treatment (A) Slit-lamp examination of the right eye showing significant improvement in corneal clarity (white arrowheads) compared to the initial visit. (B) Fluorescein staining showing resolution of epithelial damage with minimal staining (white arrowheads). (C) Endothelial specular microscopy of the right eye 13 months after referral showing measurable endothelial cells with a density of 677 cells/mm² (orange rectangle). (D) Anterior segment OCT pachymetry map demonstrating normalized corneal thickness with a central corneal thickness of 510 μm (orange rectangle), significantly reduced from 620 μm at the initial visit. OCT: optical coherence tomography

A gradual improvement in corneal edema was observed during monthly evaluations, initially in the inferior region (Figure 4A-4B). Endothelial specular microscopy was performed in seven directions. As treatment progressed, endothelial cell morphology became distinguishable. Thirteen months after referral, endothelial cell density in the central cornea was measurable at 677 cells/mm² (Figure 4C). As measured by anterior segment OCT, central corneal thickness exhibited a consistent decrease, from 620 μm at the initial visit to 510 μm 8 months after referral (Figure 4D). This reduction in thickness normalized to approximately the same level as the fellow eye (501-504 μm). Concurrently, there was an enhancement in corneal transparency, resulting in a recovery of best-corrected visual acuity to approximately 0.6-1.0 (20/33-20/20). Nine months after referral, the frequency of ganciclovir eye drops was gradually reduced to six times per day and maintained at that level thereafter.

Concerning intraocular pressure, an increase to 21 mmHg was observed during a follow-up visit at Hospital A. This resulted in administering ripasudil hydrochloride hydrate eye drops (right eye twice/day) five months after referral. Seven months after referral, the pressure remained elevated at 25 mmHg during a follow-up visit to the Kyoto Prefectural University ophthalmology department. This prompted increased betamethasone sodium phosphate eye drops to be administered three times daily in the right eye. Consequently, intraocular pressure has remained stable below 20 mmHg.

Table 1 summarizes the best-corrected visual acuity, central corneal thickness, endothelial cell density, and intraocular pressure before and after pharmacological treatment.

**Table 1:** Clinical parameters before and after antiviral treatment in the affected and fellow eyes “Before treatment” and “after treatment” refer to the time points before and 13 months after topical 0.5% ganciclovir treatment initiation. The left eye served as a reference and remained unaffected throughout the course. (*) Although a mild elevation in intraocular pressure was observed during the course of treatment, it was managed by increasing the frequency of betamethasone sodium phosphate eye drops and adding ripasudil hydrochloride eye drops, after which the intraocular pressure remained stable at or below 20 mmHg. BCVA: best-corrected visual acuity, µm: micrometers, mmHg: millimeters of mercury

Parameter	Right eye (before treatment)	Right eye (after treatment)	Left eye (reference)
BCVA decimal (Snellen)	0.06 (20/333)	0.6-1.0 (20/33-20/20)	0.8 (20/25)
Central corneal thickness (µm)	620	510	503
Endothelial cell density (cells/mm²)	Not measurable	677	2466
Intraocular pressure (mmHg)	Not measurable	<20*	19.7

Thirteen months after referral, aqueous humor testing was performed again, confirming the elimination of CMV (Figure 3B). In this case, the patient presented with unilateral, steroid-resistant corneal edema in an immunocompetent individual and experienced recurrent episodes of endotheliitis, which prompted PCR testing of the aqueous humor, leading to the detection of CMV.

## Discussion

Corneal endotheliitis is a disease that causes specific inflammation of corneal endothelial cells. This inflammation is characterized by localized corneal edema and keratic precipitates (KPs). These conditions lead to progressive corneal endothelial damage due to inflammation. The etiology is predominantly viral, with HSV, VZV, CMV, and mumps virus being the main causative agents. Among these, CMV corneal endotheliitis is a recently proposed disease concept by Koizumi [2].

The hallmark of CMV corneal endotheliitis is the presence of satellite lesions, characterized by a characteristic circular arrangement of KPs on the corneal endothelium. These satellite lesions are analogous to rejection lines, also known as Khodadoust lines, which are often observed in the aftermath of corneal transplantation. Furthermore, the presence of owl's eyes (owl-like nuclear inclusions in CMV-infected cells) has been documented in corneal endothelial cells utilizing confocal and specular microscopy [4-7]. In contrast to CMV retinitis, which manifests as an opportunistic infection in severely immunocompromised states, CMV corneal endotheliitis develops in patients without underlying diseases causing immunodeficiency. It is known to cause recurrent iridocyclitis with elevated intraocular pressure [8,9]. While the CMV antibody positivity rate among adults in Japan was previously 80-90%, recent trends show a decline from over 90% to around 60% among younger individuals [10]. A growing body of evidence, particularly from Asian countries, has highlighted that some instances previously classified as idiopathic endotheliitis, unresponsive to herpes treatment, recurrent edema following corneal transplantation for bullous keratopathy, or diagnosed as Posner-Schlossman syndrome were attributable to CMV infection [11-14].

In this case, the cause of bullous keratopathy was identified through the collection of aqueous humor and the implementation of PCR testing, leading to remission without the necessity of surgical intervention. This outcome was attained by administering 0.5% ganciclovir eye drops, resulting in the maintenance of transparent healing for a period exceeding one year following the commencement of treatment. The improvement of corneal thickness in the affected eye to the same level as the fellow eye suggests that the corneal edema has resolved. To the best of our knowledge, there have been no previous reports of cases achieving remission with pharmacological treatment alone for bullous keratopathy accompanied by severe visual impairment.

This case suggests the possibility of achieving remission with pharmacological treatment alone in bullous keratopathy with underlying CMV corneal endotheliitis. Typical bullous keratopathy is characterized by decreased endothelial cell numbers due to physical stimuli, such as surgery or iris contact, resulting in insufficient overall pump function. In contrast, the etiology of bullous keratopathy caused by CMV infection is presumed to result from decreased pump function of endothelial cells due to CMV infection of these cells. The rationale behind transparent healing in bullous keratopathy following pharmacological intervention is believed to be the reactivation of endothelial cell pump function through anti-CMV treatment.

Pharmacological treatment, in comparison with surgical treatments such as Descemet's stripping automated endothelial keratoplasty or Descemet's membrane endothelial keratoplasty, is characterized by a reduced level of invasiveness, can be administered on an outpatient basis, and has the advantage of reducing financial burden. Furthermore, in cases where surgical intervention is performed for bullous keratopathy caused by CMV-induced endothelial dysfunction without prior suspicion of CMV infection, there is a high probability of early recurrence necessitating reoperation. Consequently, screening for CMV infection and administering anti-CMV treatment based on the results is imperative before proceeding with surgical intervention [15-17]. We do not believe that all cases of CMV-related bullous keratopathy can achieve remission without surgical intervention. However, we suspect many such instances proceed to endothelial keratoplasty without anterior chamber PCR testing. Nonetheless, there are limitations to this approach, including the need for frequent administration of 0.5% ganciclovir eye drops (six to eight times daily) during the active phase of CMV corneal endotheliitis, which depends on the patient's adherence to eye drop administration, and the limited number of facilities equipped to perform viral PCR on aqueous humor.

Furthermore, the ganciclovir eye drops were prepared in-house, and the number of facilities capable of prescribing them is limited. It is recommended that patients with CMV corneal endotheliitis continue 0.5% ganciclovir eye drops two to four times daily to prevent recurrence, even after the endotheliitis has subsided [2]. For patients demonstrating poor adherence to eye drop administration, a countermeasure such as hospitalization for ganciclovir infusion treatment, accompanied by education on proper eye drop administration, could be considered.

The extant literature suggests that the majority of cases of CMV corneal endotheliitis manifest as unilateral [17]. Consequently, unilateral bullous keratopathy should prompt consideration of a CMV infection, necessitating active viral PCR testing of aqueous humor. In cases of CMV corneal endotheliitis that have progressed to bullous keratopathy, the corneal endothelial surface is often not observable, and diagnostic findings are not readily obtained through slit-lamp microscopic examination. In this particular instance, the corneal edema was so substantial at the time of examination that the presence of coin lesions or rejection-like KPs could not be confirmed, making it challenging to strongly suspect CMV corneal endotheliitis based solely on anterior segment findings. According to the diagnostic criteria for CMV corneal endotheliitis established by the Idiopathic Corneal Endotheliitis Research Group in 2012 [17], this case was classified as an atypical case.

## Conclusions

This case exemplifies that unilateral bullous keratopathy may have underlying viral etiologies, particularly CMV infection, that are amenable to pharmacological intervention. A diagnostic approach incorporating aqueous humor PCR testing can identify treatable causes, potentially obviating the need for more invasive surgical procedures. In instances where viral testing is positive for CMV, appropriate antiviral therapy with ganciclovir should be promptly initiated and continued with meticulous and long-term monitoring. In the cases where there is no observable improvement in the patient's condition despite the implementation of pharmacological interventions or where viral testing returns negative results, the recommendation is to proceed with corneal endothelial cell transplantation. For institutions lacking PCR testing capabilities, referral to specialized centers is recommended to ensure comprehensive diagnosis and optimal treatment selection. The successful treatment of CMV-related bullous keratopathy in this case may expand treatment options for patients with similar conditions beyond the traditional surgical paradigm.
